# Bridging the Gap: The Importance of Non-technical Skills in Cardiology for Enhanced Patient Care and Team Performance

**DOI:** 10.7759/cureus.75460

**Published:** 2024-12-10

**Authors:** Faiq Farooq, Shabee U Hassan, Fahad Farooq, Faizan Farooq, Muhammad Samee Khan Tareen

**Affiliations:** 1 Internal Medicine, Portsmouth Hospitals University NHS Trust, Portsmouth, GBR; 2 Cardiology, Portsmouth Hospitals University NHS Trust, Portsmouth, GBR; 3 Neurology, Cork University Hospital, Cork, IRL; 4 Intensive Care Unit, Pakistan Kidney and Liver Institute, Lahore, PAK; 5 Acute Medicine, Portsmouth Hospitals University NHS Trust, Portsmouth, GBR

**Keywords:** cardiology, communication, decision-making, healthcare costs, leadership, non-technical skills, situational awareness, stress management, teamwork, t-notechs

## Abstract

Cardiology, a high-acuity medical specialty, has traditionally emphasised technical expertise, often overshadowing the critical role of non-technical skills (NTS). This imbalance stems from the historical focus on procedural competence and clinical knowledge in cardiology training and practice, leaving a significant gap in the development of crucial interpersonal and cognitive abilities. However, emerging evidence highlights the significant impact of NTS on patient outcomes, team dynamics, and overall healthcare efficiency. This review explores the importance of NTS in cardiology whilst drawing parallels with other high-stakes fields, such as aviation, surgery, and emergency medicine, which have successfully integrated NTS frameworks. These fields are particularly relevant due to their similar requirements for rapid decision-making under pressure, team-based approaches, and the potential for severe consequences in case of errors. Key NTS domains relevant to cardiology include leadership, such as in guiding cardiac teams effectively during procedures and emergencies; cooperation and resource management, such as focusing on optimising team performance and resource allocation in cardiac care units; communication and interaction, such as clear and concise information exchange during complex cardiac interventions; assessment and decision-making such as making critical choices in time-sensitive situations; situational awareness (SA) that maintains a comprehensive understanding of the patient's condition and surrounding environment; and coping with stress, which involves managing personal and team stress during and after high-pressure situations. The integration of NTS into cardiology practice is essential for improving patient care, reducing healthcare costs, and preventing clinician burnout. Despite the recognition of NTS' importance, there is a notable lack of structured training and validated assessment tools in cardiology. This absence poses a significant challenge to the implementation of NTS, as it hinders the systematic development and evaluation of these crucial skills amongst cardiologists. For instance, whilst technical skills in procedures like angiography are rigorously assessed, there is limited formal evaluation of NTS, for instance, a cardiologist's ability to lead a team effectively during a cardiac emergency. This review advocates for the development and implementation of NTS training and evaluation methods suitable for cardiology, emphasising the need for a balanced skill set that complements technical proficiency with social and cognitive abilities. Future research should focus on adapting existing tools or developing new ones to address the unique challenges of cardiology teams and foster safer, more effective patient care. Specific areas for investigation include developing cardiology-specific NTS assessment tools, creating structured NTS training programmes for cardiology fellows, evaluating the impact of NTS training on patient outcomes in cardiac care, and exploring the relationship between NTS proficiency and technical skill performance in cardiology procedures. By addressing these research gaps and developing targeted NTS interventions, cardiology can foster a more comprehensive approach to professional development, ultimately leading to improved patient care and healthcare efficiency in this critical field.

## Introduction and background

Cardiology, a high-acuity medical specialty, has traditionally placed significant emphasis on technical expertise as the pathway to excellence. As a complex field with a heavily interventional focus, technical skills often take precedence, frequently overshadowing the critical role of non-technical skills (NTS). This imbalance manifests in cardiology training programmes and practice, where procedural competence and clinical knowledge are prioritised, leaving a substantial gap in the development of crucial interpersonal and cognitive abilities. For instance, while cardiology fellows are rigorously assessed on their ability to perform procedures like angiography, there is limited formal evaluation of their capacity to lead a team effectively during a cardiac emergency. However, growing evidence supports the importance of developing social and cognitive abilities, as career progression in cardiology requires competencies that extend well beyond technical acumen. 

The aeronautical and business sectors were early adopters in recognising the importance of NTS, incorporating these skills to enhance team dynamics, decision-making, and safety [1]. Following their lead, surgical specialties adopted NTS from the aviation model, tailoring these frameworks to address the unique demands of the operating room [2]. Similarly, acute medical fields have incorporated NTS in their training, with anaesthesia using the Anaesthetists' Non-technical Skills (ANTS) [3] and emergency medicine recognising a modified version known as a tool for NTS for trauma (T-NOTECHS) as one of the top NTS assessment tools [4,5]. These areas are especially pertinent to cardiology because similar to cardiology, they demand quick decision-making in high-pressure situations, emphasise teamwork, and carry significant risks if mistakes occur. Despite the growing recognition of NTS in healthcare, there is a notable paucity of NTS training opportunities in cardiology specialties [6]. This lack of structured training and validated assessment tools poses a significant challenge to the implementation of NTS in cardiology. Barriers to NTS adoption in cardiology include the persistence of old myths about the primacy of technical skills, economic restraints, and a lack of guidelines specifically addressing NTS in cardiac care.

Emerging evidence points towards a direct relation between subpar NTS and unfavourable patient outcomes [7]. In cardiology, poor communication and teamwork during complex procedures like percutaneous coronary interventions can lead to delays in treatment, misinterpretation of critical information, and increased risk of complications. These NTS deficiencies not only impact patient care but also contribute to inefficiency and cost in cardiology practice. In the UK, it is estimated that poor NTS cost the National Health Service (NHS) approximately £1 billion due to protracted hospital stays [8]. 

NTS encompass a broad range of attributes, and by examining the overlap between the three most recognised tools in healthcare, modified NTS scale (NOTECHS), T-NOTECHS, and team emergency assessment measure (TEAM), we can better understand how these skills manifest in cardiology. Leadership in cardiology involves guiding cardiac teams effectively during complex procedures and emergencies, such as coordinating a response to an acute myocardial infarction. Cooperation and resource management are crucial for optimising team performance and ensuring effective resource allocation, especially in high-stress situations, such as managing multiple critically ill patients in a cardiac care unit.

Clear and concise communication is vital for ensuring accurate information exchange during intricate cardiac interventions, such as during coronary artery bypass graft surgery, where every detail can impact patient outcomes. Decision-making and assessment are central to making timely, critical choices in situations like determining the best intervention for a patient in cardiogenic shock, where the window for intervention is often narrow. Finally, situational awareness (SA) and stress management are essential for maintaining a comprehensive understanding of both the patient’s condition and the broader environment, while also managing the personal and collective stress that can arise from a high-pressure job.

Together, these NTS help shape a balanced, effective approach to cardiology, where technical expertise is complemented by strong leadership, collaboration, and decision-making in the face of time-sensitive, high-stakes situations.

## Review

This review explores existing knowledge about the nuances of NTS within cardiology and aims to highlight the paramount role of NTS within the specialty’s high-intensity clinical environment, along with advocating for a structured evaluation and training to balance technical expertise with the social and cognitive skills necessary for effective, safe practice in cardiology.

Non-technical skills (NTS)

*Leadership* 

Cardiologists face a diverse range of challenges, both within the specialty and in collaboration with closely related fields. These challenges encompass clinical, administrative, and logistical aspects, each demanding strategic navigation and effective problem-solving. Among the key attributes essential for overcoming these obstacles, leadership stands out as vital. Leadership skills in a cardiologist facilitates guiding multidisciplinary teams, making critical decisions, and driving patient-centred care in complex situations. Leadership directly impacts specific aspects of cardiology practice. In team efficiency, effective leaders can coordinate the efforts of various specialists during complex procedures like percutaneous coronary interventions, reducing delays and improving outcomes. In critical care decision-making, strong leadership is crucial for rapid and accurate assessments during cardiac emergencies, such as cardiac arrest due to cardiac tamponade. Enhanced clinical and financial outcomes and patient safety have a direct relation with effective leadership within the medical profession [9]. Although current physician leadership programmes seem to improve self-assessed knowledge and expertise, most of these do not test system-level outcomes such as improvement in quality indicators for disease management. Therefore, there remains a gap for meaningful leadership development programmes that can translate into patient care improvement [10].

The lack of leadership training in cardiology stems from several factors. Traditionally, cardiology training has focused heavily on technical skills and clinical knowledge, often at the expense of leadership development. The complexity of cardiovascular cases and the rapid pace of technological advancements in the field can overshadow the need for NTS training. Moreover, the lack of investment in leadership development is a broader healthcare problem especially in the NHS where the challenge of serving large populations often means that quantity can take precedence over quality of care, marginalising the need for NTS training. This challenge is particularly evident in cardiology, where the workload on cardiologists is consistently on the rise.

Furthermore, the current healthcare system often does not prioritise or allocate resources for leadership training in cardiology fellowships. The Core Cardiovascular Training Statement 4 (COCATS 4) from the American College of Cardiology (ACC) has only recently recognised the importance of leadership skills, indicating a shift in perspective but also highlighting the historical lack of emphasis on this area.

Emphasis should be placed on teaching positive and active leadership styles like those carrying supportive and collaborative attributes [11]. An effective leader in cardiology would possess emotional intelligence, enabling them to navigate complex social networks in clinical settings, manage working relationships efficiently, and inspire and influence others. It has been observed that negative and passive leadership attributes are associated with poor patient outcomes, resource depletion, and burnout amongst physicians [12]. Negative leadership styles could create a toxic environment where team members are afraid to speak up about potential issues, compromising patient safety.

Cooperation and Resource Management 

In the complex and intense environment of cardiology, effective cooperation and resource management are crucial for delivering optimal patient care. These skills involve working collaboratively with multidisciplinary teams, efficiently allocating resources, and coordinating care across different healthcare settings. 

Cooperative and collaborative care programmes between general practitioners (GPs) and cardiologists have been shown to be associated with improved quality across a broad range of indicators, including pharmacotherapy and vaccination rates [13]. If formalised, these programmes have the potential to establish structured cooperation between primary care physicians and cardiologists, which have led to tangible benefits in managing cardiovascular patients. For instance, such collaborations have resulted in more appropriate use of anticoagulation therapy in atrial fibrillation patients and improved adherence to guideline-directed medical therapy for heart failure [14]. This eventually leads to lower emergency service utilisation and hospitalisations and reduced consultation frequencies with GPs and specialists. These outcomes underscore the importance of structured cooperation between primary care physicians and cardiologists in managing cardiovascular patients. 

Effective teamwork, resource management, adaptability, and team orientation are well recognised traits for effective delivery of care in a complex socio-technical system such as a cardiovascular intensive care unit (CVICU) [15]. For example, in managing a patient on extracorporeal membrane oxygenation (ECMO), effective teamwork involves not only the coordination between physicians, nurses, and perfusionists but also their interaction with the ECMO machine and monitoring systems. This socio-technical perspective ensures that both human factors and technological aspects are considered in optimising patient care and safety in the high-stakes CVICU environment.

*Communication and Interaction* 

Effective communication and interaction are indispensable skills for cardiologists, playing a vital role in improving patient care, outcomes, and satisfaction. These skills encompass not only the ability to convey complex medical information clearly but also the capacity to listen actively, empathise, and engage patients in shared decision-making. One-on-one training on five key skills; making eye contact, asking open-ended questions, using reflective statements, employing empathetic statements, and soliciting patient concerns - shows significant improvements in the interactions cardiologists have with their patients [16,17]. 

The impact of improved communication skills extends beyond patient satisfaction, effective communication skills in the form of reflective and empathic statements by cardiologists are associated with higher patient participation [18]. This is subsequently linked to better recall of information, improved adherence to treatment, and enhanced clinical outcomes. For instance, in post-myocardial infarction care, effective communication can significantly improve adherence to medication regimens and therefore improve long-term outcomes of these procedures.

The low-socioeconomic (SES) subset of patients are particularly affected by lack of this skill set, in that they voice fewer concerns, and express fewer utterances overall compared to higher-SES patients, hence highlighting the critical importance of effective communication in context of SES disparities [19]. This disparity stems from various factors, including lower health literacy, language barriers, and reduced access to health information amongst low-SES populations. Cardiologists need to be particularly adept at tailoring their communication style to bridge these gaps, ensuring that all patients, regardless of their SES, receive and understand critical information about their cardiovascular health.

The importance of communication skills is particularly evident in discussions about prognosis, where individualised, context-specific communication is preferred by both patients and cardiologists to build a foundation of trust and rapport [20]. This is especially crucial in cardiology, for instance, in the case of advanced cardiac failure, where discussions often involve complex treatment options, lifestyle changes, and long-term management.

Recognising the critical nature of these skills, there have been efforts to incorporate communication training into cardiology education. The Beckwith Institute's CardioTalk programme, for instance, is a workshop designed to improve communication through short didactic sessions followed by interactions with standardised patients. It focuses on competencies such as giving bad news, defining goals of care, responding to emotion, and supporting religious beliefs. This programme has shown promising results, with participants reporting improved preparedness for leading difficult conversations. The COCATS 4 has only recently recognised the importance of communication skills which marks the change in outlook whilst underscoring the historical neglect of this critical area.

Teaching communication micro-skills to cardiologists managing seriously ill patients demonstrates its positive impact on physicians by making them relatively comfortable in dealing with difficult and complex conversations [21]. There is a need for more structured, comprehensive communication skills training programmes that are integrated into cardiology fellowship curricula and continue throughout a cardiologist's career.

Assessment and Decision-Making 

In cardiology, the ability to assess situations and make decisions extends beyond clinical aspects to include various non-clinical matters that significantly impact patient care. Being able to identify the importance of non-clinical factors especially those that influence medical decisions, such as patient’s socioeconomic status, quality of life, values, and beliefs [22], is a key NTS particularly for cardiologists. Like many other NTS, decision-making and clinical judgement have also received emphasis by COCATS 4 as part of the training curriculum in the US.

Clinical and non-clinical assessment and decision-making are closely intertwined. For instance, a patient's socioeconomic status may influence their ability to adhere to a complex medication regimen or attend regular follow-up appointments, which in turn affects clinical decision-making about treatment options. Similarly, a patient's cultural beliefs may impact their willingness to undergo certain procedures, requiring cardiologists to adapt their approach.

Clinical and non-clinical assessment and decision-making involves resource allocation, whilst considering the prospect of incurring extra cost to the healthcare system by unnecessary investigations [23], and the rising cost of sequelae of poor cardiovascular health [24]. Cardiologists often face such decisions about the use of expensive diagnostic tools or treatments in a resource-constrained environment. For example, a cardiologist might need to weigh the cost of a cardiac MRI against its potential diagnostic value, considering both the immediate expense to the healthcare system and the long-term benefits for the patient. This decision becomes more complex when considering the rising costs of sequelae of undiagnosed cardiac disorders. Often personal factors such as previous negative experiences, patients’ expectations, and managing uncertainty in diagnosis influence a cardiologist’s decision-making and assessment [25]. 

Another important area of decision-making relates to ethical considerations. Cardiologists frequently encounter situations that require balancing medical benefits against patient autonomy or quality of life considerations. For instance, deciding whether to recommend a high-risk surgical intervention for an elderly patient with multiple comorbidities and determining the appropriateness of continuing aggressive treatment in end-stage heart failure are common ethical dilemmas. These decisions often involve weighing the principle of beneficence against non-maleficence and respecting patient autonomy. The efficacy of decision-making can be improved if a more individual-centred approach is employed considering factors like patient’s own wishes about their future care and their beliefs and values [26]. This approach aligns with the concept of shared decision-making (SDM), which is increasingly recognised as crucial in cardiology practice. SDM involves collaborating with patients to create mutually agreed-on care plans that align with patients' values, preferences, and life circumstances [27].

By developing these assessment and decision-making skills, cardiologists can significantly enhance the quality of care they provide, leading to a more efficient healthcare delivery. The integration of clinical expertise, resource considerations, ethical awareness, and patient-centred approaches is essential for effective decision-making in modern cardiology practice.

Situational Awareness (SA) 

SA is a dynamic state of conscious perception that arises through the interplay of several cognitive processes, including working memory, long-term memory, intellectual adaptability, proactive focus, and guideline understanding. It also involves the use of mental pathways formed through extensive clinical training and repeated exposure to relevant situations [28]. In the context of cardiology, SA enables practitioners to anticipate potential complications, make timely interventions, and optimise patient outcomes. Working memory helps cardiologists rapidly process and integrate multiple data points during complex procedures, such as angioplasty, allowing them to anticipate potential complications like arrhythmias or vessel perforation.

Improving SA by implying methods such as changes in escalation pathways, evening rounds, visual management boards, and daily huddles have shown significant improvement in code events and emergency transfers amongst paediatric cardiology patients [29]. This can be employed in cardiology settings such as in CVICU, cardiologists can foster SA within multidisciplinary teams through structured communication tools like Situation, Background, Assessment, Recommendation (SBAR) during handovers. Implementing daily multidisciplinary rounds with a visual management board can ensure all team members have a shared understanding of each patient's condition and care plan.

The importance of SA in cardiology extends beyond individual patient care to team performance. As outlined by the Canadian Medical Protective Association (CMPA), team SA refers to 'the collective understanding of the evolving situation, involving knowledge of tasks as well as team roles and responsibilities'. CMPA recommends cultivating a supportive culture allowing effective two-way communication and psychological well-being of the staff in order to improve SA of a team [30]. Cardiologists can actively contribute to creating a supportive culture by modelling open communication, encouraging questions from junior staff, and regularly debriefing after critical events to identify areas for improvement.

The NHS Wales Situational Awareness Toolkit emphasises three levels of SA: perception of elements in the current situation, comprehension of the current situation, and projection of future status. It outlines the significant role of systematically gathering, analysing, and disseminating information within clinical teams. This method holds particular importance in cardiology, a field often characterised by intricate patient cases and swiftly evolving clinical situations. For perception, cardiologists can use standardised checklists during patient assessments to ensure comprehensive data gathering. Comprehension can be enhanced through regular case discussions where team members share their interpretations of clinical data. Projection skills can be developed through hypothetical discussions or organised simulations, where cardiologists practice anticipating and responding to potential complications.

Coping With Stress 

Stress not only affects a cardiologist’s personal well-being but also significantly impacts the quality of patient care they provide; therefore, the ability to cope with stress effectively is particularly important in cardiology, given the high-stakes nature of the specialty and the potential for burnout [31]. 

Effects of mental stress on coronary events is a well-recognised fact [32], underscoring the importance of stress management not only for patients but also for cardiologists who regularly deal with high-stress situations. The Medscape Cardiologist Lifestyle Report 2017 identified only 56% of cardiologists to be happy ‘outside of work’ in comparison to the 69% of urologists [33]. Factors associated with burnout amongst cardiologists include extensive administrative duties, long working hours, the growing reliance on digital systems in practice, limited control over work, and pressures to fulfil certification requirements [31]. 

Burnouts have been seen affect various metrics of patient-care including reduced standard of treatment, elevated frequency of healthcare mistakes, diminished patient contentment, lowered efficiency, and increased staff attrition amongst clinicians [34]. 

To mitigate physician burnout factors, a combined strategy from the individuals, healthcare organisations and medical societies is required. This should include a multitiered approach providing support and burnout preventative interventions ranging from primordial prevention to secondary prevention [34]. This approach considers the complex interplay between individuals, interpersonal relationships, institutions, communities, and public policy [35]. At the individual level, interventions such as mindfulness training, stress management techniques including regular exercise, and strategies for achieving work-life balance can be beneficial. These individual-focused interventions have been shown to be effective in reducing burnout amongst healthcare professionals [36].

Organisationally, implementing flexible work schedules, reducing administrative burdens, and fostering a positive workplace culture such as by introducing cultural transformation practices are essential steps. The Substance Abuse and Mental Health Services Administration (SAMHSA) has outlined several evidence-based organisational interventions to prevent and reduce burnout amongst behavioural health provided [35]. These include the availability, responsiveness, and continuity (ARC) intervention, participatory workplace intervention, and multicomponent intervention, which have been shown to increase job satisfaction, decrease work-related exhaustion, and improve organisational commitment [35].

Systemic changes are also critical; addressing issues in medical education, licensing processes, and certification requirements can help create a more supportive environment for cardiologists. The National Academy of Medicine's National Plan for Health Workforce Well-Being emphasises the need for a collaborative approach involving health systems, academic institutions, professional societies, and government bodies [37].

Specific organisational interventions have proven effective in reducing burnout amongst healthcare professionals, including cardiologists. For example, adopting team-based care models can help distribute workload more evenly and improve efficiency. Providing dedicated time for research and professional development allows clinicians to engage in fulfilling activities outside their clinical responsibilities. Additionally, peer support programmes and mentorship opportunities can foster a sense of community and shared experience amongst clinicians [36,38]. Kelsey et al. highlighted the benefits of being part of a community, such as a virtual book club, in reducing burnout [36].

By addressing burnout through these multifaceted approaches, healthcare organisations can enhance both cardiologist well-being and the overall quality of patient care. As emphasised in recent studies, a culture of openness and support that encourages practitioners to seek help when needed is essential for sustainable improvements in healthcare professionals' well-being [36,38].

Evaluation of NTS 

NTS are particularly critical in cardiology due to the complex, fast-paced, and high-pressure nature of the specialty. Procedures such as percutaneous coronary interventions (PCI), valve replacements, and managing cardiac arrest situations require cardiologists to make rapid, high-stakes decisions whilst coordinating with multidisciplinary teams. The growing recognition of teamwork and communication in cardiology settings, such as in multidisciplinary cardiac surgery teams, catheterisation laboratories, or during emergencies like ST-elevation myocardial infarction (STEMI), underscores the need for effective NTS training and assessment.

Video-based assessment tools, such as T-NOTECHS [5], offer the advantage of allowing for more objective and repeatable assessments. However, applying these tools in cardiology may present challenges such as technical barriers, patient privacy concerns, and contextual differences in care settings. Non-video tools like TEAM have gained recognition in emergency medicine [39] but may face limitations in cardiology due to the different team dynamics and interactions involved.

Real-world applications of NTS assessment tools in cardiology could include settings like the cardiac catheterisation laboratory, intensive care unit (ICU), or heart failure clinics. These environments demand a unique combination of technical expertise and critical decision-making under time pressure. For example, during a complex PCI procedure, cardiologists must not only demonstrate technical proficiency but also effectively communicate with team members, maintain SA, and make rapid decisions based on changing patient conditions.

The Team Emergency Assessment Measure (TEAM) could potentially be adapted for use in acute cardiac emergencies, such as during resuscitation efforts in the cardiac ICU. However, it would need to be tailored to account for the specific roles and interactions of cardiology team members, including interventional cardiologists, cardiac surgeons, and specialised cardiac nurses.

To address the lack of a valid NTS assessment tool in cardiology, collaborative efforts between cardiologists, educational researchers, and simulation experts are needed to tailor existing tools or develop new ones specific to cardiac care. This could involve adapting tools like T-NOTECHS or TEAM to incorporate cardiology-specific scenarios and team dynamics.

Integrating NTS into current cardiology training programmes could involve incorporating simulation-based assessments that evaluate both technical skills and NTS during high-fidelity scenarios. By emphasising NTS in cardiology education and assessment, the field can better prepare practitioners to navigate the complexities of modern cardiac care, ultimately improving patient outcomes and team performance in high-pressure and high-risk cardiac care settings.

We notice that T-NOTECHS, NOTECHS, and TEAM share common parameters in their assessment of NTS, including leadership, communication, situation awareness, decision-making, and teamwork/cooperation (Table 1). 

**Table 1:** Comparison of T-NOTECHS, NOTECHS, and TEAM tools [40-42]

Aspect	Trauma non-technical skills scale (T-NOTECHS) [40]	Non-technical skills scale (NOTECHS) [41]	Team Emergency Assessment Measure (TEAM) [42]
Purpose	Evaluates non-technical skills specifically in trauma resuscitations	Assesses non-technical skills primarily in surgery	Measures non-technical skills in emergency medical teams
Domains evaluated	Leadership, cooperation/teamwork, communication, decision-making, situation awareness	Leadership, cooperation, communication, decision-making, situation awareness	Leadership, teamwork, task management
Application Area	Trauma, primarily in emergency and critical care	Surgical healthcare settings	Emergency medicine, especially resuscitation and critical care.
Assessment Method	Video-based assessment in trauma setting	Non-video, observational assessment in operating rooms	Can be used in both simulated and real-life settings

Whilst these tools have shown promise in emergency medicine, their applicability and reliability in cardiology settings remain to be explored. Future research should focus on adapting existing tools or developing new ones specifically tailored to the unique challenges and dynamics of cardiology teams. 

## Conclusions

Whilst technical proficiency remains essential in cardiology, this review underscores the role of NTS in ensuring effective, safe, and holistic patient care. Drawing from advancements in other high-acuity fields, NTS domains like leadership, communication, cooperation, SA, decision-making, and stress management prove paramount within the specialty. Evidence linking poor NTS with negative patient outcomes and increased healthcare costs highlights the need for structured NTS training and assessment in cardiology. Although current tools, such as T-NOTECHS and TEAM, have demonstrated efficacy in emergency and trauma settings, there remains a critical gap in validated, cardiology-specific NTS assessment methods. Future research should aim to adapt and develop such tools to meet the unique demands of cardiology, fostering a balanced skill set that combines technical expertise with vital social and cognitive competencies. Techniques such as simulation-based training and team drills can deepen our understanding of how to achieve an optimal balance between technical and NTS, helping us determine the appropriate emphasis each skill requires for effective and holistic practice.
